# Effects of Synthetic Acaricides and *Nosema ceranae* (Microsporidia: Nosematidae) on Molecules Associated with Chemical Communication and Recognition in Honey Bees

**DOI:** 10.3390/vetsci7040199

**Published:** 2020-12-08

**Authors:** Martín Pablo Porrini, Paula Melisa Garrido, María Laura Umpiérrez, Leonardo Pablo Porrini, Antonella Cuniolo, Belén Davyt, Andrés González, Martín Javier Eguaras, Carmen Rossini

**Affiliations:** 1 Centro de Investigación en Abejas Sociales (CIAS), Instituto de Investigaciones en Producción Sanidad y Ambiente (IIPROSAM), Consejo Nacional de Investigaciones Científicas y Técnicas (CONICET), Universidad Nacional de Mar del Plata (UNMdP), Funes 3350, Mar del Plata 7600, Argentina; pmgarrid@mdp.edu.ar (P.M.G.); leoporrini@gmail.com (L.P.P.); antocuniolo@gmail.com (A.C.); meguaras@mdp.edu.ar (M.J.E.); 2 Laboratorio de Ecología Química, Facultad de Química, Universidad de la República Uruguay, Montevideo 11800, Uruguay; mlumpierr@fq.edu.uy (M.L.U.); bdavyt@gmail.com (B.D.); agonzal@fq.edu.uy (A.G.); crossini@fq.edu.uy (C.R.)

**Keywords:** *Apis mellifera*, nosemosis, acaricides, primer pheromone, hydrocarbon profiles, survival

## Abstract

Acaricides and the gut parasite *Nosema ceranae* are commonly present in most productive hives. Those stressors could be affecting key semiochemicals, which act as homeostasis regulators in *Apis mellifera* colonies, such as cuticular hydrocarbons (CHC) involved in social recognition and ethyl oleate (EO) which plays a role as primer pheromone in honey bees. Here we test the effect of amitraz, coumaphos, *tau*-fluvalinate and flumethrin, commonly applied to treat varroosis, on honey bee survival time, rate of food consumption, CHC profiles and EO production on *N. ceranae*-infected and non-infected honey bees. Different sublethal concentrations of amitraz, coumaphos, *tau*-fluvalinate and flumethrin were administered chronically in a syrup-based diet. After treatment, purified hole-body extracts were analyzed by gas chromatography coupled to mass spectrometry. While *N. ceranae* infection was also shown to decrease EO production affecting survival rates, acaricides showed no significant effect on this pheromone. As for the CHC, we found no changes in relation to the health status or consumption of acaricides. This absence of alteration in EO or CHC as response to acaricides ingestion or in combination with *N. ceranae*, suggests that worker honey bees exposed to those highly ubiquitous drugs are hardly differentiated by nest-mates. Having determined a synergic effect on mortality in worker bees exposed to coumaphos and Nosema infection but also, alterations in EO production as a response to *N. ceranae* infection it is an interesting clue to deeper understand the effects of parasite-host-pesticide interaction on colony functioning.

## 1. Introduction

Current research efforts looking for causes of honeybee colony losses agree on a common hypothesis: the causes are multifactorial due to combined effects of different stressors acting as drivers of lethal or sublethal effects on colony members. These effects include diseases and xenobiotics as two of most likely causes.

Regarding chemical substances, research has focused mainly on the effects of agricultural pesticides; however, there are a growing number of studies referring to colony disruptions produced by in-hive pesticides. This is the case of acaricides, which are periodically incorporated into apiaries suffering varroosis worldwide, representing a huge source of wax and food contamination [1,2].

Among honey bee diseases, *Nosema* spp. infections have been intensively studied, focusing mainly on *Nosema ceranae* as a relatively new microsporidiosis affecting *Apis mellifera*, which has generated a continuously reviewed body of information [2,3,4,5,6]. Further than causing a large list of effects at individual and colony level, *N. ceranae* (recently proposed to be reclassified as *Vairimorpha ceranae* [7]), was also studied in combination with different chemical stressors, evidencing interactions with agricultural pesticides [5]. However, just a few studies have focused on the interaction of *N. ceranae* with acaricides [8,9].

The combination of chemicals and diseases in honeybee colonies clearly represents a challenge to the homeostasis, an equilibrated status depending on, among other factors, volatile chemical compounds (semiochemicals) which are social key regulators in *Apis mellifera* [10,11,12,13]. Cuticular hydrocarbons (CHC) and ethyl oleate (EO) are among those semiochemicals, which are also potentially involved in social immunity [14,15].

In the hive, olfactory discrimination mediated by CHC plays a critical role in self-segregation to differentiate between castes, hive of origin and physiological health statuses [16,17,18,19,20]. Regarding nosemosis, changes in the hydrocarbon profiles of the bees have been reported as a consequence of the infection [21,22], however is still discussed if behavioral changes are associated with these CHC alterations [23].

On the other hand, EO, identified as the major component of a primer pheromone that acts as a worker inhibitory factor in honey bees [24,25], is involved in the nurse-to-forager transition, which can be altered by means of *N. ceranae* infection [15,26].

Although the referred homeostatic mechanisms are essential for social functioning and are feasible to be altered by different stressors, the studies on the impact of diseases and chemicals on honeybee pheromones and recognition cues are scarce. This background gave rise to our hypothesis that nosemosis and acaricides can impact on chemical communication cues. Therefore, in the present work, we addressed a laboratory approach to study the effect of different synthetic acaricides and *Nosema ceranae* on honeybee survival, EO production and CHC profiles.

## 2. Materials and Methods

### 2.1. Chemicals

The acaricides coumaphos, amitraz, flumethrin and *tau*-fluvalinate and the internal standards (arachidic acid methyl and tridecane) were obtained from Sigma-Aldrich, Saint Louis, MO, USA; and HPLC-grade solvents from Machery-Nagel, Bethlehem, PA, USA or LiChrosolv were used. Concentrations were chosen based on previous data reporting the presence of these pesticides in pollen and honey. *Tau*-fluvalinate was detected at a concentration of 750 ppb in honey [27] and of 487.2 ppb in pollen [28]: the selected dose was approximately the average between those values (666 ppb). In the case of coumaphos, 2020 ppb were found in honey [29] and 5800 ppb in pollen [30], so the selected dose was approximately the average (3333 ppb). The maximum detection in honey for amitraz was 555 ppb and for flumethrin was 1 ppb [31].

### 2.2. Experimental Procedure

Experiments were performed using hybrid honey bees of *Apis mellifera mellifera* and *Apis mellifera ligustica* from hives located at the experimental apiary of the Social Bees Research Centre (38°10′06″ S, 57°38′10″ W), Mar del Plata, Argentina. Colonies used to obtain brood combs were selected based on their low abundance of *N. ceranae* spores (<5 × 10^5^ spores/bee estimated in forager bees using the technique described by Fries [32] and their low prevalence of *Varroa destructor* Anderson and Trueman (Acari: Varroidae) mites, according to the season. The mite prevalence was evaluated by the natural mite fall method [33] and the rates of phoretic mite infestation [34]. Furthermore, neither of the colonies used to obtain the imagoes presented any visible clinical symptoms of other diseases (i.e., American foulbrood, chalkbrood or viruses).

**Figure 1:**
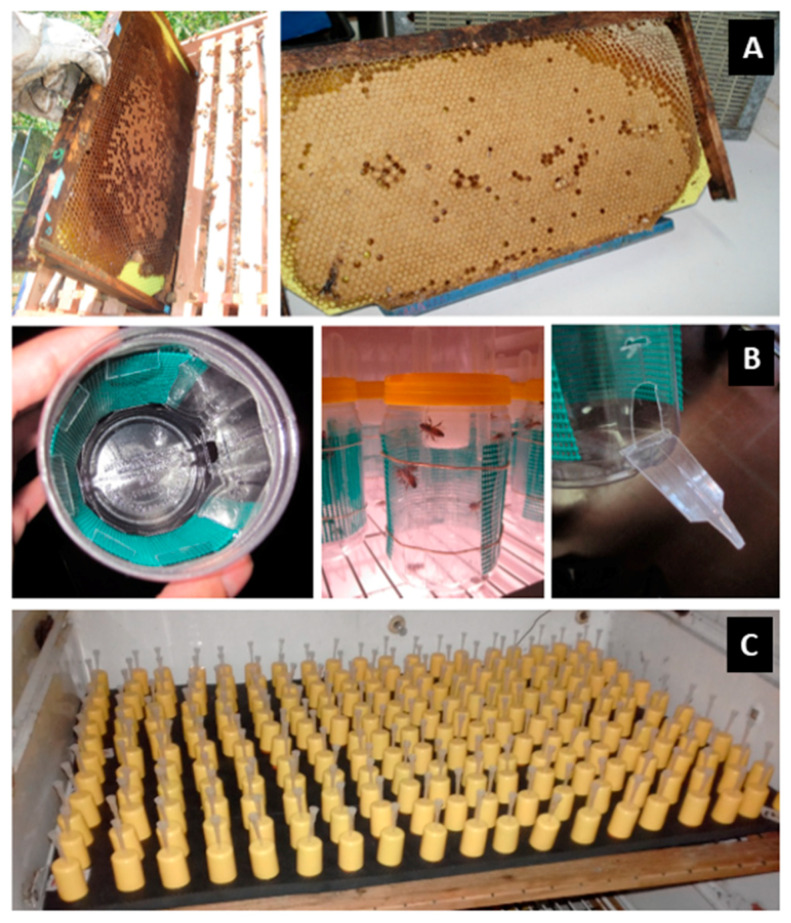
(**A**) Langstroth combs with virgin beeswax over plastic foundation as initial matrix. (**B**) Confinement devices made using plastic flasks, plastic mesh for ventilation, perforated cap for feeding devices and removable lateral door. The dimensions of each cage were 150 × 70 mm^2^ (height × diameter). (**C**) Individual inoculation devices (Porrini et al., 2013).

To avoid the presence of long lasting acaricide residues, commonly found in new commercial beeswax [35], plastic foundations covered with a thin layer of virgin wax were used. These foundations were placed in the selected colonies three months before building the assays began and serve as brood combs. For every assay, frames of sealed brood from one hive were transported in thermic boxes and maintained under incubator conditions until imagoes emerged (34 °C; 60% RH). Newly emerged honey bees were placed in groups of 200–300 in wooden cages (11 × 9 × 6 cm^3^) with a plastic mesh until they were randomly assigned to disposable treatment cages. For each these included in the experimental design, groups of 2-day-old honey bees (N = 50 imagoes/group) were caged in transparent and ventilated plastic flasks (900 cm^3^) with inputs for gravity feeding devices and a removable side door (Figure 1), specifically designed following the criteria for good rearing of adult honey bees [36]. The honey bees were maintained in the flasks and placed in an incubator (28 °C; 30% RH) during the assays. In all experiments, each group consisted of five experimental replicates (flasks) unless otherwise noted.

Honey bees were fed ad libitum with syrup (sucrose-water solution; 2:1 w:v) and beebread (stored pollen) collected from combs of the same hive of origin and not treated with acaricides during the previous 9 months. Besides, water and one of the different treatment diets (diluted in the sucrose syrup) or control syrup were also provided throughout the assays. Except for pollen (replaced weekly), all diet items were replaced daily.

Three experiments were sequentially run to assess the effect of infection status and acaricide ingestion on the recorded variables.

#### 2.2.1. Experiment I—Effect of *Nosema ceranae* Infection and Ethanol

This assay was designed to study the effect of *N. ceranae* infection on EO production and CHC profiles, but also to test the effect of ethanol (3.2%) on those two variables since other experiments with acaricides were performed adding that solvent into the diet.

To achieve the infection, two-day-old honey bees were individually fed according to a technique improved to avoid trophallaxis and possible contamination between confined individuals and to homogenize the stress during inoculation procedures [37]. The infective dose consisted of 10 µL of 50% sugar solution including freshly extracted spores of *N. ceranae* at a concentration of 1 × 10^5^ spores/µL. Spores were obtained from heavily infected forager bees and were concentrated and cleaned from other midgut contents using a triangulation technique [38]. The infection with *N. ceranae* was confirmed by PCR analysis at the end of the assays [39]. After artificial inoculation, honey bees were confined in plastic flasks as described in Section 2.2. Four experimental groups were established according to infection and diet administered for 13 days: Infected honey bees with ethanolic diet (INF+EtOH); infected honey bees without ethanolic diet (INF); non-infected honey bees with ethanolic diet (CTRL+EtOH) and non-infected honey bees without ethanolic diet (CTRL).

#### 2.2.2. Experiment II—Effect of Acaricides

The experiment was designed to simulate realistic conditions of exposure to acaricides to analyze its effect on the recorded variables (Section 2.1). To prepare the diets, acaricidal compounds were dissolved in EtOH 96% and then added to sugar syrup. As previously explained, honey bees were fed for 13 days with solutions of coumaphos (3333 ppb), amitraz (555 ppb), flumethrin (1 ppb) or *tau*-fluvalinate (666 ppb). Therefore, five experimental groups were established: control honey bees under ethanolic diet (CTRL); honey bees fed on coumaphos (COUM); honey bees fed on amitraz (AMI); honey bees fed on flumethrin (FLUM) and honey bees fed on *tau*-fluvalinate (FLUV).

#### 2.2.3. Experiment III—Effect of the Combination of *Nosema ceranae* Infection and Coumaphos

The aim of this assay was to study the effect of *N. ceranae* infection combined with an orally administered concentration of coumaphos on EO production and CHC. To perform *N. ceranae* infection, workers were confined and treated following the protocol detailed in Experiment I (Section 2.2.1). After inoculation, two groups of honey bees (infected and non-infected) were supplied with water, beebread and one of three different syrup diets ad libitum for 13 days. The diets consisted of sugar syrup including EtOH 3.2% or coumaphos (3333 ppb) diluted in EtOH 3.2%. Then, four experimental groups were established: Infected honey bees that received syrup diet (INF); infected honey bees on syrup with coumaphos (INF+Coum); non-infected honey bees on syrup (CTRL) and non-infected honey bees on syrup with coumaphos (CTRL+Coum).

### 2.3. Recorded Variables

During the assays, different parameters such as survival and substance consumption were recorded, but also, different analyses were performed on sacrificed honey bees, such as parasite development and pheromones and CHC quantifications.

#### 2.3.1. Survival and Diet Consumption

Honey bees without movement response after mechanical stimulation were recorded as dead and removed from containers on a daily basis. In addition, gravity feeders were weighted every day to estimate consumed amounts. Diet evaporation was checked in feeding devices and inside flasks without honey bees to correct the consumed volumes.

#### 2.3.2. *Nosema ceranae* Development

In Experiments I and III, 13 days post-infection, 15–20 honey bees per replicate were analyzed to individually quantify the parasite development. The digestive tract of the honey bees was dissected, keeping only the midguts, which were stored at −20 °C until quantification. The number of spores was individually estimated with a hemocytometer under a light microscope [40].

#### 2.3.3. EO and CHC Quantification

In all assays, after a period of 13 days, pools of five honey bees/replicates were sacrificed and stored for EO and CHC quantification. The extraction protocol was adapted from a previous report [15]. Briefly, after sacrifice, whole-body extracts were prepared in hexane (1.6 mL) with the addition of 200 μL of two internal standard solutions (arachidic acid methyl ester at 5 ng/μL, and tridecane at 50 ng/μL). Subsequently, samples were crushed (2 min, RT) and centrifuged (20 min, 4 °C, 2000 rpm). The supernatants were applied onto SPE-Silica columns (Alltech, Lexington, KY, USA, Clean TM 1000 mg/8 mL) and eluted with mixtures of hexane and ethyl ether. The first fraction (3 mL of hexane-ethyl ether; 98.5:1.5) contained the CHC. The fraction containing fatty acid esters (including EO) was eluted with hexane-thyl ether (94:6), was left to evaporate under a liquid nitrogen stream, and 1 µL analyzed by gas chromatography coupled to mass spectrometry (GCMS). GCMS analyses were done using a QP-2010 Plus Shimadzu acquiring mass spectra from m/z 28 to 350 in the scan mode (70 eV) on an RTX-5ms column (Restek, Bellefonte, PA, USA; 30 m × 0.25 mm i.d., 0.25 μm film thickness), operated with a constant carrier (He) flow of 1 mL/min. The temperature of the GC oven was programmed from an initial temperature of 40 °C (1 min), then heated to 300 °C at 5 °C/min, and held for 1 min. The injector temperature was 250 °C and the interphase temperature was 300 °C in GCMS analysis. Chemical characterization was performed by comparison of the mass spectra and arithmetic retention indexes calculated from data of a solution of hydrocarbons injected in the same conditions [41] to those reported in the NIST2008 [42] and SHIM2205 [41] databases and in the literature as well as by comparisons with synthetic standard in the case of EO. Quantification was done based on the relative areas of compounds compared to the areas of the corresponding internal standards.

## 3. Statistical Analyses

Statistical analyses were run on MINITAB 17.3.1 and Past (PAleontological Statistics, Oslo, Norway). In each experiment survival curves were compared among treatments by log-rank and Holm–Sidak method. Food consumption was also compared for every assay using ANOVA or Kruskal–Wallis test. Infection levels with *Nosema* spores were also compared by Mann–Whitney tests. To compare levels of EO and CHC between treatments, data was assessed for normality using Anderson–Darling procedure before subjecting it to an ANOVA/GLM and Tukey HSD tests, using as factors the dietary treatment administered (“DIET”) and the condition of “infected” or “not-infected” (HEALTH STATUS) where corresponded. In the case of CHC profiles principal component analyses (PCA) were also run on scaled and centered data. The significance level used for every assay was 0.05.

## 4. Results

### 4.1. Experiment I—Effect of Nosema ceranae Infection on EO Production and CHC Profile

#### 4.1.1. *Nosema ceranae* Development

At the end of the experiment, no spores were found in midguts from non-infected honey bees. Midguts extracted from infected honey bees fed with or without ethanol reached a mean ± SE of 1.5 × 10^6^ ± 0.4 × 10^6^ spores/bee (INF+EtOH), and 2.0 × 10^6^ ± 0.5 × 10^6^ spores/bee (INF). No significant differences were found between spore counts (Mann–Whitney rank sum test; U statistic = 79; T = 119; *p* = 0.44).

#### 4.1.2. Survival and Diet Consumption

**Figure 2:**
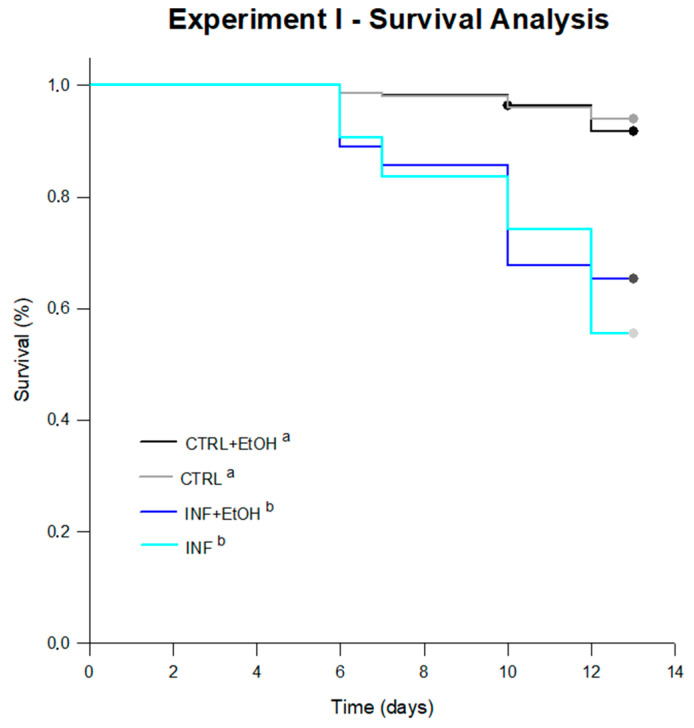
Survival curves for bees under treatments in Experiment I. Significant differences were found between survival curves after 13 days of experiment (log-rank test; statistic: 103.491; df = 3; *p* < 0.001). Significant differences between treatments are indicated with different letters (pairwise multiple comparison procedures Holm–Sidak method; *p* = 0.05). The average survival (estimated by the test) for each treatment was 12.79 days for CTRL, 11.45 days for INF, 12.77 days for CTRL+EtOH and 11.46 days for INF+EtOH.

Significant differences were found between survival curves (Figure 2) after 13 days of experiment (log-rank test; statistic: 103.491; df = 3; *p* < 0.001). Infected honey bees with or without EtOH included in their diets, showed lower survival than non-infected honey bees (pairwise multiple comparison procedures Holm–Sidak method; *p* = 0.05). Overall cumulative mortality reached 40 ± 7% (pooled data, mean ± SD) for infected honey bees and a 7 ± 2% for non-infected ones. The average survival (estimated by the test) for each treatment was 12.79 days for CTRL, 11.45 days for INF, 12.77 days for CTRL+EtOH and 11.46 days for INF+EtOH. Consumption rates were not different between treatments, with average consumption (grouping all treatments) of 31 ± 5 mg/bee/d (ANOVA, df = 3, F = 1.478, *p* = 0.258).

#### 4.1.3. Ethyl Oleate

**Figure 3:**
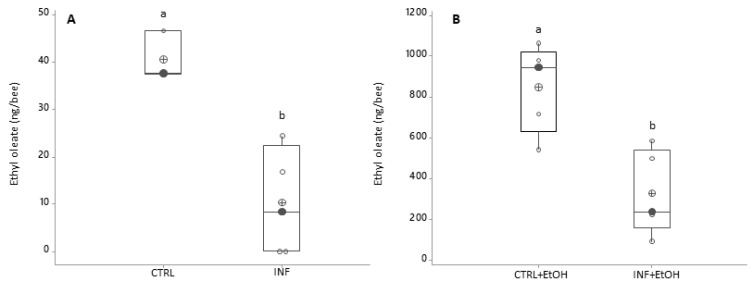
Ethyl oleate variation as a function of infection status and ethanol consumption (black circles are the median values; crossed circles are the mean values and open circles are individual data). *X*-axis labels are as follows: (**A**) Treatments without ethanolic diet: not-infected honey bees (CTRL); infected honey bees (INF); (**B**) Treatments with ethanolic diet: not-infected honey bees (CTRL+EtOH) and infected honey bees (INF+EtOH). Different letters indicate significant differences: differences between honey bees with different diets and health status were significant (ANOVA-GLM: F_1,16_ = 47.99 for diet, *p* < 0.001; F_1,16_ = 11.43 for health status, *p* = 0.005 and F_1,16_ = 9.06 for the interaction diet*health status, *p* = 0.01).

*Nosema*-infected honey bees showed a decrease in EO compared to the level detected in non-infected bees (10 ± 6 and 41 ± 3 ng/bee, respectively; Figure 3A). This reduction, was registered even if honey bees had ingested ethanol in the diet: infected individuals fed on ethanol-supplemented diet showed an average production ± SD of 330 ± 91 ng/bee compared to non-infected honey bees on the same diet (851 ± 96 ng/bee); Figure 3B; ANOVA-GLM: F_1,16_ = 47.99 for diet, *p* < 0.001; F_1,16_ = 11.43 for health status, *p* = 0.005 and F_1,16_ = 9.06, for the interaction diet/health status, *p* = 0.01).

#### 4.1.4. Cuticular Hydrocarbons

**Figure 4:**
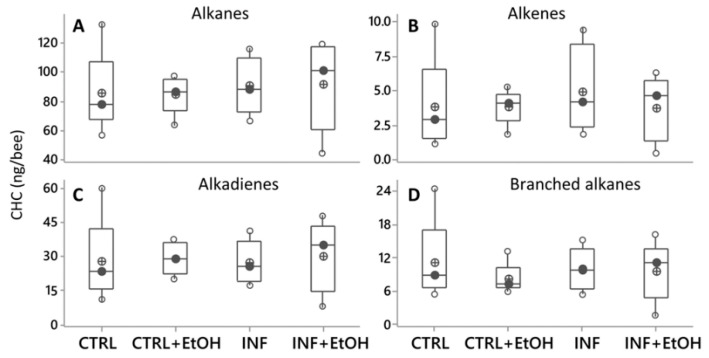
Cuticular hydrocarbons (CHC) profiles shown as compound families (**A**–**D**), sowing variation as a function of diets. Black circles are the median values; crossed circles are the mean values and open circles are individual data. No differences were found for any of the compound families related to diet (with or without ethanol) or health (with or without *Nosema* infection; ANOVA (GLM), *p* > 0.05 in all cases. *X*-axis labels are as follows: not-infected honey bees without ethanolic diet (CTRL); infected honey bees without ethanolic diet (INF); not-infected honey bees with ethanolic diet (CTRL+EtOH) and infected honey bees fed on ethanolic diet (INF+EtOH).

The PCA (var-covar matrix) on the 39 quantified CHC did not find any obvious grouping between honey bees with different health statuses (not-infected vs. *Nosema* infected) or diets (syrup vs. syrup+ethanol). The PCA on all data from the four categories showed that the data were well explained by three components (Component 1:72%; Component 2:11%, Component 3:6% Joliffe cut-off 2.64) and the CHC that better explained the variance in the data (higher loadings) were mainly the same as in all other experiments. No differences were found either on individual CHC when analyzing them by ANOVA (GLMs, all *p* values > 0.05) or when analyzing them by chemical class (alkanes, alkenes, branched alkanes and alkadienes, Figure 4A–D, Figure S1, Table S1). In addition, different models built by discriminant analyses did not pass validation (permutation tests, *p* > 0.05), indicating an absence of a pattern related to CHC from honey bees under different treatments.

### 4.2. Experiment II–Effect of Acaricides on EO Production and CHC Profile

#### 4.2.1. Survival and Diet Consumption

**Figure 5:**
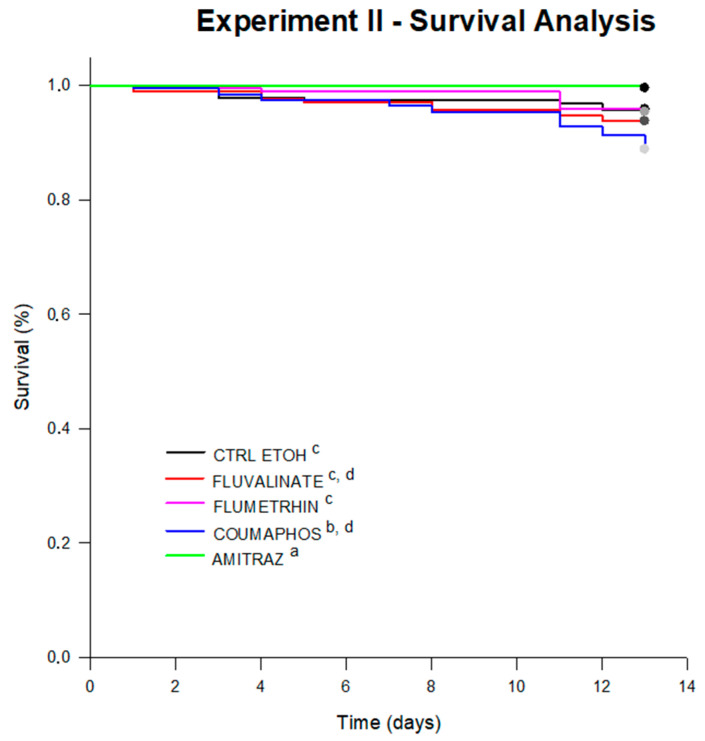
Survival curves for bees under treatments in Experiment II. Significant differences were found between survival curves after 13 days of experiment (log-rank test; statistic: 16,957; df = 4; *p* = 0.002). Significant differences between treatments are indicated with different letters (pairwise multiple comparison procedures Holm–Sidak method; *p* < 0.05). The average survival (estimated by the test) for each treatment was 12.71 days for CTRL (control diet), 12.83 days for FLUM (flumethrin), 12.61 days for FLUV (fluvalinate), 13.00 days for bees under AMI (amitraz) and 12.57 days of survival for bees under COUM (coumaphos treatment).

After 13 days of diet consumption, honey bees under flumethrin or fluvalinate treatment (exhibiting average survival estimated by the test of 12.83 days and 12.61 days, respectively), did not suffer mortality different from bees under the control diet (12.71 days on avg.). However, bees under coumaphos treatment (12.57 days of survival on avg.) suffered a significant reduction in survival (log-rank test; *p* = 0.047). Furthermore, honey bees treated with amitraz showed a higher probability of survival compared to the control (13.00 day survival on avg.; log-rank test; *p* = 0.01) and the other treatments (Figure 5).

Similar food consumption was found between acaricide-treated bees and the control (21 ± 1 mg/bee/d on avg.; ANOVA, df = 5, F = 0.516, *p* = 0.761).

**Figure 6:**
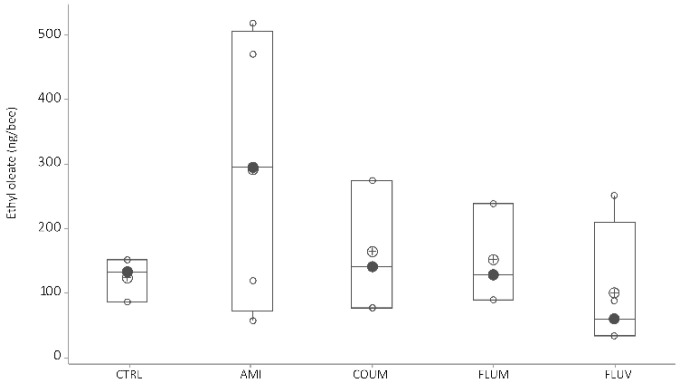
Ethyl oleate variation as a function of acaricide treatment (black circles are the median values; crossed circles are the mean values and open circles are individual data). Differences between honey bees with different diets are not significant (*p* > 0.05, Mann–Whitney tests, see text for further details). *X*-axis labels are as follows: control honey bees under ethanolic syrup diet (CTRL+EtOH); honey bees feed on amitraz in ethanolic syrup diet (Amit+EtOH); honey bees fed on coumaphos in ethanol syrup diet (Coum+EtOH); honey bees feed on flumethrin in ethanol syrup diet (Flum+EtOH) and honey bees on fluvalinate in ethanol diet (Fluv+EtOH).

Ethyloleate—When honey bees were fed with diets including acaricides the level of ethyloleate did not vary compared to the level of honey bees fed with no acaricides (Figure 6, ANOVA-GLM: F_4,16_ = 1.09, *p* = 0.404; Kruskal–Wallis test, H = 2.45, df = 4, *p* = 0.65).

#### 4.2.2. Cuticular Hydrocarbons

**Figure 7:**
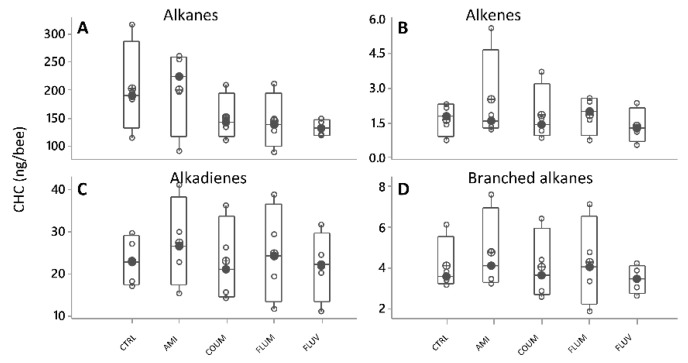
Cuticular hydrocarbons (CHC) profiles shown as compound families (**A**–**D**), showing variation as a function of diet (black circles are the median values; crossed circles are the mean values and open circles are individual data). No significant differences were found (ANOVA, GLMs, *p* > 0.05 in all cases). *X*-axis labels are as follows: control honey bees under ethanolic syrup diet (CTRL+EtOH); honey bees feed on amitraz in ethanolic syrup diet (Amit++EtOH); honey bees fed on coumaphos in ethanol syrup diet (Coum+EtOH); honey bees fed on flumethrin in ethanol syrup diet (Flum+EtOH) and honey bees on fluvalinate in ethanol diet (Fluv+EtOH).

After a PCA (var-covar matrix) on the 39 quantified CHC, any obvious groupings were found between honey bees fed with different diets (control syrup, amitraz, coumaphos, flumethrin and fluvalinate). The PCA on all data from the six categories showed that the data was well explained by two components (Component 1:91%; Component 2:3%, Joliffe cut-off 15.63) and the CHC that better explained the variance in the data (higher loadings) were again the same as in all other experiments (the alkanes n-heneicosane, n-tricosane, n-pentacosane, n-heptacosane and n-nonacosane, and the alkenes, 9-hentriacontene and triacontene). Besides, different models built by discriminant analyses did not pass validation (permutation tests, *p* > 0.05), indicating an absence of patterns related to CHC from honey bees under different treatments. No differences were either found in individual CHC when analyzing them by ANOVA (GLMs, *p* > 0.05 in all cases) or when analyzing them by chemical class (alkanes, alkenes, branched alkanes and alkadienes (Figure 7A–D, Figure S1, Table S2).

### 4.3. Experiment III—Effect of Nosema ceranae Infection and Coumaphos on EO Production and CHC Profile

#### 4.3.1. *Nosema ceranae* Development

No spores were found in non-infected honey bees (CTRL and CTRL+Coum). Infected honey bees from different dietary treatments (INF and INF+Coum) showed no statistical differences among them (Mann–Whitney rank sum test; U statistic = 71; *p* = 0.63) and reached a mean ± SE of 1.4 × 10^6^ ± 0.5 × 10^6^ spores/bee (INF), and 2.1 × 10^6^ ± 1.1 × 10^6^ spores/bee (INF+Coum).

#### 4.3.2. Survival and Diet Consumption

**Figure 8:**
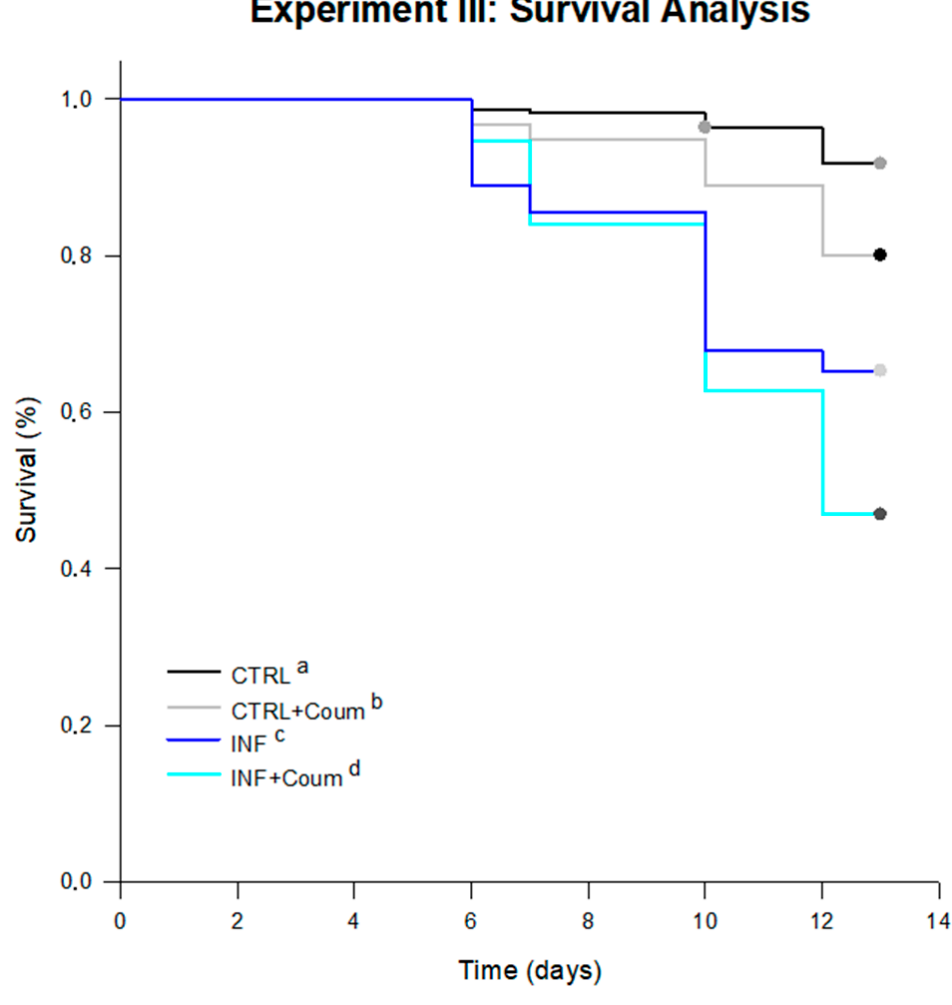
Survival curves for bees under treatments in Experiment II. Significant differences were found between survival curves for every treatment (log-rank test; statistic: 92.85; df = 3; *p* < 0.001). The average survival times, estimated by the test in days for every treatment were: 12.77 days for non-infected honey bees fed on syrup diet (CTRL), 12.39 days for non-infected honey bees fed on syrup diet and coumaphos (CTRL+Coum), 11.46 days for infected honey bees fed on syrup diet (INF); 11.19 days for infected honey bees fed on syrup diet and coumaphos (INF+Coum). Significant differences between treatments are indicated with different letters (pairwise multiple comparison procedures Holm–Sidak method; *p* < 0.05).

Significant differences were found between survival curves for every treatment (log-rank test; statistic: 92.85; df = 3; *p* < 0.001; pairwise multiple comparison of survival curves Holm–Sidak method; overall significance level = 0.05). The average survival times, estimated by the test in days for every treatment were: 11.46 days for infected honey bees fed on syrup diet (INF); 11.19 days for infected honey bees fed on syrup diet and coumaphos (INF+Coum); 12.77 days for non-infected honey bees fed on syrup diet (CTRL) and 12.39 days for non-infected honey bees fed on syrup diet and coumaphos (CTRL+Coum). Infected honey bees showed significantly lower survival than non-infected ones, independently of the inclusion of coumaphos in the diet (Figure 8). In absence of infection, coumaphos included in diet caused a significant reduction in the survival time compared to the control treatment. Furthermore, a combined effect of “*Nosema* infection” and “coumaphos ingestion” was detected in “survival” since INF+Coum treatment showed a significantly lower survival rate than other treatments.

Consumption rates were mainly similar between treatments with an average value (±SD) of 37 ± 15 mg/bee/day. The only treatment showing larger consumption rate than the others was the one combining coumaphos and *Nosema* infection, from day 8 post-infection onwards (Kruskal–Wallis one way analysis of variance on ranks, H = 12,462; df = 5; *p* = 0.029).

#### 4.3.3. Ethyloleate

**Figure 9:**
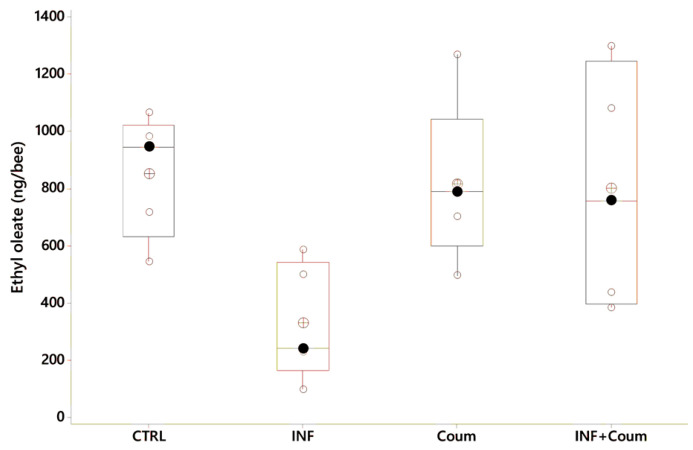
Ethyl oleate variation as a function of acaricide consumption and health status (black circles are the median values; crossed circles are the mean values and open circles are individual data). *X*-axes labels are as follows: non-infected honey bees without ethanolic diet (CTRL); non-infected honey bees on ethanolic syrup diet (CTRL+EtOH); non-infected honey bees on ethanolic and coumaphos syrup diet (CTRL+EtOH+Coum); infected honey bees without ethanolic diet (INF); infected honey bees on ethanolic syrup diet (INF+EtOH) and infected honey bees on ethanolic and coumaphos syrup diet (INF+EtOH+Coum). Differences between honey bees with different diets are significant (F_2,25_ = 19.03, *p* < 0.001) but not with different health status (F_1,25_ = 3.54, *p* = 0.075).

In this experiment the ANOVA (GLM) on ethyloleate levels showed a significant variation as a function of the treatment (F_3,18_ = 3.45, *p* < 0.04), with infected bees exhibiting the lower EO levels (Figure 9, post-GLM comparisons, Fisher pairwise comparisons).

#### 4.3.4. Cuticular Hydrocarbons

**Figure 10:**
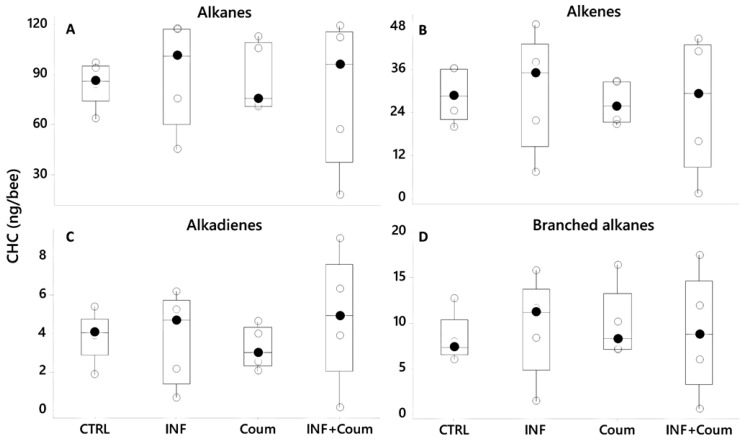
Cuticular hydrocarbons (CHC) profiles shown as compound families (**A**–**D**), showing variation as a function of diet and health status (black circles are the median values; crossed circles are the mean values and open circles are individual data). No significant differences were found (*p* > 0.05 in all cases). *X*-axes labels are as follows: non-infected honey bees without ethanolic diet (CTRL); non-infected honey bees on ethanolic syrup diet (CTRL+EtOH); non-infected honey bees on ethanolic and coumaphos syrup diet (CTRL+EtOH+Coum); infected honey bees without ethanolic diet (INF); infected honey bees on ethanolic syrup diet (INF+EtOH) and infected honey bees on ethanolic and coumaphos syrup diet (INF+EtOH+Coum).

A PCA (var-covar matrix) on the 39 quantified CHCs showed that the data was well explained by three components (Component 1: 75%; Component 2:11%, Component 3:6%; Joliffe cut-off 33,528) and the CHCs that better explained the variance in the data (higher loadings) were like the ones previously found: mainly linear CHC (n-tricosane, n-pentacosane, n-heptacosane, n-nonacosane and n-hentriacontane) and the alkenes 9-heptacosene, 9-nonacosene, 9-ticosane and 9-hentriacontene. The PCA distribution showed non-grouping patterns among honey bees on different treatments; moreover, all of the models built by discriminant analyses did not pass validation (permutation tests, *p* > 0.05), indicating an absence of pattern related to CHC from honey bees under different treatments. ANOVAs (GLM) on CHC compound families (alkanes, alkenes, alkadienes and branched alkanes) showed non-significant effect correlated to the treatments for the four compound families (*p* > 0.05, Figure 10, Figure S1, Table S3). No significant effects were found either for the 39 individual CHC computed (GLMs, *p* > 0.05 in all cases) related to the treatments.

## 5. Discussion

After performing different assays, to study the effect of *Nosema* disease and chemical stressors on pheromone production, cuticular fingerprint and survival, we can highlight two major results: a reduction in EO production in infected workers and no alterations in CHC as response to acaricides ingestion or under a combination of coumaphos ingestion and *N. ceranae* infection.

### 5.1. Effect of Nosema ceranae Infection

In line with previous findings, our experimentation showed that *Nosema*-infected honey bees exhibited lower survival than non-infected ones, confirming the negative impact of the disease caused by *Nosema ceranae*, even when administering a diet based on fresh pollen under laboratory conditions [43,44,45]. Furthermore, the infection itself did not alter syrup consumption when comparing daily food intake, similarly to a previous report [46]. Moreover, in the cited work, an increase in *Nosema* spp. infection was observed when feeding honey bees with 5% ethanol in syrup, different levels to our finding in which *Nosema* spores reached similar counts in control and ethanol-fed honey bees, indicating that the consumption of ethanol at 3.2% does not affect *Nosema* development, at least, combined with a fresh pollen diet. The result on consequences of ethanol ingestion in infected bees is a relevant topic since it is commonly used to dissolve some substances studied as therapeutical control of *N. ceranae* (i.e., propolis, herbal extracts).

The infection itself decreased EO production in both control and ethanol-fed honey bees, diminishing approximately 3 to 4 times the amount measured, specifically from 41 ng/bee to 10 ng/bee without ethanol incorporated in the diet, and from 851 ng/bee to 330 ng/bee with an ethanolic diet. The production of EO is energetically costly; its low levels could be explained in part by the negative effects of *N. ceranae* on energy metabolism and the disruption of the epithelial cells of the midgut [47,48,49,50,51]. However, this decrease in EO levels is contrary to previous reports by Dussaubat et al. [15,26], where non-infected worker honey bees exhibited lower EO levels than infected ones (approximately 100 ng/bee vs. 600 ng/bee, respectively). Perhaps, this difference might arise from methodological differences; Dussaubat et al. [15] administered a higher initial inoculum (200,000 spores/bee) to 1-day-old honey bees and found a maximum level of 25 million spores. In our study, 3-day-old honey bees were infected with 100,000 spores/bee, and a maximum of 6–7 million spores were observed. However, since the degree of parasite development was found to be correlated with EO production [15], the relatively low development of the parasite in our assay may explain overall lower levels of EO, but hardly explain that infected honey bees exhibited lower EO titers than healthy (not infected) ones. Additionally, Dussaubat et al. [15] administered a mixed inoculum including *N. ceranae* and *Nosema apis* spores while, in our assay, *N. ceranae* was the only species inoculated. Possibly, this is another cause of differences in the EO amounts found, mainly because of the different physiological effects reported for each *Nosema* species on worker honey bees [52,53].

The methodological differences between our work and the cited ones, surely cannot by themselves completely explain the dichotomy in the results obtained about EO production, therefore, further laboratory and field experiments should assess the change of EO expression along the ontogeny of the workers, under different initial doses of spores in the inoculum. Furthermore, differences in pheromone production resulting from either *N. ceranae*, *N. apis* or both, infections, could reveal interesting results, analogous to the differences shown for cuticular hydrocarbon profiles, differentially affected by infection with those microsporidian species [22].

It has been hypothesized that immune stimulation by pathogens could result in cuticular hydrocarbon changes that may indicate to other honey bees that these individuals are suffering impairment to their health statuses. This has been confirmed for different immune response elicitors (non-replicative pathogen molecules) causing an immune challenge that resulted in hydrocarbon change [54]. Since we found no changes in the individual CHC, the CHC family (Figure 3) or the CHC overall profiles of honey bees in relation with its health status or consumption of ethanol, our data would refute this hypothesis, at least for bees who reach the infection level reported here. Nevertheless, two previous studies have shown plasticity on CHC profiles correlated with health status.

As mentioned, Murray et al. [22] demonstrated differences in the CHC profiles of young (of undefined age) honey bees infected by *N. ceranae* compared to non-infected ones, although no behavioral modification towards *Nosema*-infested nest-mates was found in the same study (this last being a current matter of discussion after the results presented by Biganski et al. [23]). Furthermore, McDonnell et al. [21] measured CHC from honey bees originated from three different hives, showing that, when comparing the overall chemical profile of two of them, infected honey bees showed differences with non-infected ones after 10 days, counting from artificial infection date. In our opinion, these partially contrasting results with previous reports might be attributed to sensitive differences in experimental procedures between assays. The most relevant of those differences, were the ones related to the age of the bees and the homogeneity of the cohort studied, but also, to some differences in the extraction methods performed to obtain the samples for the analyses of the CHC profiles. Future studies should cautiously consider the age and cohort studied, achieving analysis of the CHC profiles in marked honey bees (*Nosema* infected and non-infected), released into the hive and recovered after different periods, to understand how the parasite can affect communication cues under realistic conditions.

### 5.2. Effect of Acaricides

Exposure to pesticides, including both the ones used for agricultural pests and those administered in the hive, can substantially affect physiological pathways in honeybee workers. Specifically, the sublethal effects of many acaricides, including those caused by *tau*-fluvalinate and coumaphos on physiological functions [55], behavior [56], reproductive castes [57] and immune responses [9,58,59], have been previously reported. However, scarce antecedents are available studying the effects of chemical stressors on honey bee pheromones. In the present work, although some toxicity was found in honey bees consuming coumaphos in their diet, none of the four acaricides tested showed effects on EO titers or CHC profiles. As far as we know, the absence of effect on worker chemical communication and recognition cues, after a chronic exposure to sublethal doses of acaricides, constitutes a new report. Moreover, the absence of effect on EO production was also reported for the pesticide imidacloprid, a neonicotinoid commonly used in integrated pest management in crops [15] and frequently found in hives [28]. The cited report, coupled with our results regarding acaricides, conceivably suggests a general pattern which needs to be corroborated.

It cannot be ruled out that the concentrations tested here (chosen from available data about contaminated honey and pollen stored in hives [29,30] may not have been enough to trigger some effects on the measured variables. Therefore, further experiments designed to emulate a post-treatment scenario, including the study of the combined effect of two or more acaricides at higher sublethal doses could yield different results [60]. A further consideration comes from the quality of the administered diet, since it has been demonstrated that pollen-based diets reduce the sensitivity of workers to pesticides exposure [61]; therefore, providing honey bees with high-quality nutrition, as we did, may have attenuated the effect of pesticides.

### 5.3. Effect of Nosema ceranae Infection and Coumaphos

It has been demonstrated that there is a possibility of joint negative effects of *Nosema* spp. and chemical stressors such as imidacloprid, fipronil or thiacloprid, impacting directly on honey bee survival, social immunity or sometimes promoting parasite development [8,43,44,62,63,64,65]. Previous results revealed no evidence of combined effects of *Nosema ceranae* and acaricides on honeybee survival and in a variety of immune related genes [9]. In agreeing with that, we found no evidence of synergistic effects of both coumaphos and *Nosema* infection EO production or CHC profiles. However, compared with survival data obtained by Garrido et al. [9] until day 9 of consumption, we found a decrease in survival rate caused by the coumaphos diet from day 10 onwards, evidencing a possible cumulative effect of the drug in the worker honey bee organism. Furthermore, the combination of both stressors (coumaphos and *Nosema* disease) significantly decreased the survival rate, suggesting a synergy between both factors.

The accumulation of lipophilic acaricides on beeswax is well known [35,66,67,68]. During our assays, honey bees only received acaricide exposure as imagoes while pre-imaginal stages were developed in combs with a presumed absence of acaricide residuals (see M & M section). Therefore, it will be interesting to analyze the effect of exposure to sublethal doses even during larval development in future designs.

Since in-field studies demonstrated a complex relationship between acaricides and colony homeostasis as well as different effects of acaricides on the progression of nosemosis disease [69,70,71], it is relevant to delve deep into the subjacent causes involved in that complexity by means of realistic field or semi-field assays.

## 6. Conclusions

In the laboratory assays, we found a reduction in EO production in infected young workers, even with low or medium *N. ceranae* spore development, which could possibly influence the nurse-to-forager transition in a hive. Beyond differences in our results with those ones found in previous works, reporting significant alterations in EO production as a response to *N. ceranae* infection can contribute to deeply understanding the effects of the disease on colony functioning. Since a high prevalence of the disease could be found in a hive, reaching percentages over 50% of individuals infected [72,73], any alteration derived from the infection at individual level could be magnified at colony level especially, in sensitive periods during the year or under stressing situations.

On the other hand, not finding evidence of alteration in CHC as response to acaricides ingestion or in combination with *N. ceranae*, is an innovative result that could suggest the impossibility of workers to recognize nest-mates exposed to sublethal doses of those highly ubiquitous drugs.

Finally, we encourage performing of toxicological-parasitological studies under field-realistic conditions since those are necessary to reveal the influence of *Nosema*-infected honey bees on intra-colony interactions, to better understand the relationship between stressors and social immunity, but also to deepen knowledge about how chemical substances can influence the parasitism in a population.

## Supplementary Materials

The following are available online at https://www.mdpi.com/2306-7381/7/4/199/s1, Figure S1: PCA graphs for experiments I (A), II (B) and III (C), Table S1: CHC from honeybees in Experiment I, Table S2: CHC from honeybees in Experiment II, Table S3: CHC from honeybees in Experiment III.
