# Supplementary material for: Effects of Synthetic Acaricides and Nosema ceranae (Microsporidia: Nosematidae) on Molecules Associated with Chemical Communication and Recognition in Honey Bees
Source: Vet Sci. 2020 Dec 8;7(4):199. doi: 10.3390/vetsci7040199 (PMC7768465; doi:10.3390/vetsci7040199)

veterinary sciences

Article

Effects of Synthetic Acaricides and *Nosema ceranae* (Microsporidia: Nosematidae) on Molecules Associated with Chemical Communication and Recognition in Honey Bees

Martín Pablo Porrini [1,*], Paula Melisa Garrido [1], María Laura Umpiérrez [2], Leonardo Pablo Porrini [1], Antonella Cuniolo [1], Belén Davyt [2], Andrés González [2], Martín Javier Eguaras [1] and Carmen Rossini [2]

[1] Centro de Investigación en Abejas Sociales (CIAS), Instituto de Investigaciones en Producción Sanidad y Ambiente (IIPROSAM), Consejo Nacional de Investigaciones Científicas y Técnicas (CONICET), Universidad Nacional de Mar del Plata (UNMdP), Funes 3350, Mar del Plata 7600, Argentina; pmgarrid@mdp.edu.ar (P.M.G.); leoporrini@gmail.com (L.P.P.); antocuniolo@gmail.com (A.C.); meguaras@mdp.edu.ar (M.J.E.)

[2] Laboratorio de Ecología Química, Facultad de Química, Universidad de la República Uruguay, Montevideo 11800, Uruguay; mlumpierr@fq.edu.uy (M.L.U.); bdavyt@gmail.com (B.D.); agonzal@fq.edu.uy (A.G.); crossini@fq.edu.uy (C.R.)

* Correspondence: mporrini@mdp.edu.ar; Tel./Fax: +54-223-4752426 (int 223)

Received: 6 October 2020; Accepted: 3 December 2020; Published: date

Supplementary Material

Table S1. Suppl. Material: CHC from honeybees in Experiment I.

	Compund ID	Compound Class	Retention Time	Retention index	CTRL	CTRL+EtOH	INF	INF+EtOH
1	IS (tridecane)	IS	8.934	1300				
2	NI	NI	12.138	1476	0.3 ± 0.1	0.2 ± 0.1	0.3 ± 0.1	0.3 ± 0.1
3	NI	NI	12.96	1521	0.3 ± 0.1	0.3 ± 0	0.4 ± 0.2	0.2 ± 0.1
4	NI	NI	15.994	1687	0.4 ± 0.1	0.3 ± 0	0.5 ± 0.1	0.4 ± 0.1
5	n-nonadecane	alkane	19.874	1900	3.9 ± 0.6	1.9 ± 0.3	1.9 ± 0.6	1.8 ± 0.5
6	NI	NI	20.351	1937	0.1 ± 0	0.1 ± 0	0.1 ± 0.1	0.1 ± 0
7	n-eicosane	alkane	21.147	2000	0.1 ± 0	0.1 ± 0	0.2 ± 0.1	0.1 ± 0
8	n-heneicosane	alkane	23.572	2100	4.9 ± 0.7	4.1 ± 0.4	2.9 ± 0.7	3 ± 0.7
9	n-docosane	alkane	25.324	2200	0.6 ± 0.2	0.7 ± 0.2	0.5 ± 0.2	0.4 ± 0.1
10	tricosadiene	alkadiene	26.542	2272	0.2 ± 0.1	0.1 ± 0	0.2 ± 0.1	0.2 ± 0.1
11	9-tricosene	alkene	26.589	2274	2.6 ± 0.8	5.1 ± 2.7	2.2 ± 0.5	2.4 ± 0.6
12	7-tricosene	alkene	26.704	2281	0.3 ± 0.1	0.4 ± 0.1	0.3 ± 0.1	0.3 ± 0.1
13	n-tricosane	alkane	27.023	2300	17.2 ± 2.2	17.4 ± 2.2	16.8 ± 1.6	17 ± 2.8
14	n-tetracosane	alkane	28.643	2400	1.4 ± 0.5	1.1 ± 0.1	1.6 ± 0.6	1.3 ± 0.3

15	pentacosadiene	alkadialkene	29.775	2472	0.7 ± 0.3	0.6 ± 0.2	0.9 ± 0.3	0.8 ± 0.3
16	9-pentacosene	alkene	29.831	2475	4.1 ± 1.1	4.6 ± 0.7	4.5 ± 0.7	4.6 ± 1
17	7-pentacosene	alkene	29.945	2482	1.5 ± 0.4	1.4 ± 0.2	2.3 ± 0.6	2 ± 0.5
18	n-pentacosane	alkane	30.223	2500	19.3 ± 2.7	21.3 ± 1.9	24.1 ± 2	23.8 ± 3
19	methylpentacosanes	branched alkane	30.736	2534	1.3 ± 0.5	1 ± 0.2	1.1 ± 0.3	1.1 ± 0.3
20	n-hexacosane	alkane	31.724	2600	1.5 ± 0.6	1.3 ± 0.2	1.9 ± 0.7	1.7 ± 0.5
21	heptacosadiene	alkadiene	32.643	2662	0.2 ± 0.1	0.2 ± 0	0.4 ± 0.3	0.1 ± 0
22	9-heptacosene	alkene	32.844	2676	2.7 ± 1.3	2.9 ± 0.5	2.6 ± 0.4	3.2 ± 0.8
23	7-heptacosene	alkene	32.955	2684	0.8 ± 0.4	0.8 ± 0.1	0.9 ± 0.1	1.1 ± 0.3
24	n-heptacosane	alkane	33.195	2700	20.2 ± 2.3	22.9 ± 1.9	24.6 ± 1.8	24.8 ± 3.6
25	methylhetptacosanes	branched alkane	33.641	2732	5.7 ± 1.6	4.3 ± 0.6	5.2 ± 1	5 ± 1.2
26	n-octacosane	alkane	34.586	2800	0.9 ± 0.4	0.6 ± 0.1	0.9 ± 0.3	0.7 ± 0.2
27	nonacosadiene	alkadiene	35.444	2863	2 ± 1	2.2 ± 0.4	2.6 ± 0.9	1.5 ± 0.4
28	9-nonacosene	alkene	35.652	2878	1.7 ± 0.7	2.1 ± 0.3	1.6 ± 0.3	2.2 ± 0.6
29	7-nonacosene	alkene	35.708	2882	1.1 ± 0.4	0.8 ± 0.1	1 ± 0.3	0.9 ± 0.2
30	n-nonacosane	alkane	35.952	2900	9.9 ± 1.7	9 ± 1.1	10.2 ± 1.5	10.4 ± 2.1
31	methylnonacosanes	branched alkane	36.359	2931	3.2 ± 1	2.4 ± 0.3	2.8 ± 0.5	2.8 ± 0.7
32	n-triacontane	alkane	37.259	3000	0.3 ± 0.1	0.2 ± 0	0.2 ± 0.1	0.2 ± 0.1
33	hentriacontadiene	alkadiene	38.069	3064	0.2 ± 0.1	0.2 ± 0	0.2 ± 0.1	0.2 ± 0.1
34	9-hentriacontene	alkene	38.241	3077	3.6 ± 0.7	2.9 ± 0.5	3 ± 0.6	3.2 ± 0.9
35	7-hentriacontene	alkene	38.328	3084	3.4 ± 0.7	2.6 ± 0.4	3 ± 0.6	3.2 ± 0.8
36	n-hentriacontane	alkane	38.533	3100	4.6 ± 1.2	3.9 ± 0.7	4.5 ± 0.7	5.5 ± 1.4
37	methylhentriacontane	branched alkane	38.901	3115	1 ± 0.4	0.6 ± 0.1	0.8 ± 0.1	0.8 ± 0.2
38	tritriacontadiene	alkadiene	40.496	3179	0.6 ± 0.2	0.5 ± 0.1	0.7 ± 0.2	0.9 ± 0.3

39	X-triacontene	alkene	40.738	3189	6.1 ± 2.1	5.5 ± 0.8	5.9 ± 1.1	7.1 ± 1.7
40	n-tritriacontane	alkane	41.015	3300	0.9 ± 0.5	0.3 ± 0.1	0.3 ± 0.1	0.5 ± 0.1
	Alkanes				85.7 ± 12.7	84.9 ± 5.8	90.5 ± 9.8	91.3 ± 13.9
	Alkadienes				3.8 ± 1.6	3.8 ± 0.6	5 ± 1.6	3.8 ± 1
	Alkene				27.9 ± 8.6	29.2 ± 3.3	27.4 ± 4.9	30.2 ± 7.1
	Branched alkanes				11.2 ± 3.4	8.2 ± 1.2	9.9 ± 1.9	9.7 ± 2.4
	NI				1 ± 0.2	0.8 ± 0.2	1.3 ± 0.4	0.9 ± 0.1

Table S2. Suppl. Material: CHC from honeybees in Experiment II.

	**Compund ID**	**Compound Class**	**Retention Time**	**Retention index**	**AMI**	**COUM**	**CTRL**	**FLUM**	**FLUV**
1	IS (tridecane)	IS	8.934	1300					
2	NI	NI	12.138	1476	**2.6 ± 0.3**	**2.9 ± 0.3**	**2.6 ± 0.4**	**3.2 ± 0.7**	**2.7 ± 0.4**
3	NI	NI	12.96	1521	**1.1 ± 0.4**	**2 ± 0.5**	**1.9 ± 0.7**	**1.5 ± 0.5**	**1.4 ± 0.7**
4	NI	NI	15.994	1687	**1.4 ± 0.3**	**1 ± 0.1**	**1.6 ± 0.4**	**1.1 ± 0.4**	**0.9 ± 0**
5	n-nonadecane	alkane	19.874	1900	**3.5 ± 0.7**	**2.6 ± 0.5**	**2.6 ± 0.4**	**3.1 ± 0.7**	**2.8 ± 0.5**
6	NI	NI	20.351	1937	**1.5 ± 0.2**	**1.5 ± 0.3**	**1.5 ± 0.4**	**1.7 ± 0.3**	**1.3 ± 0.1**
7	n-eicosane	alkane	21.147	2000	**0.8 ± 0.2**	**0.9 ± 0.2**	**1 ± 0.2**	**0.7 ± 0.3**	**0.8 ± 0.2**
8	n-heneicosane	alkane	23.572	2100	**4.6 ± 0.6**	**4.3 ± 0.5**	**3.7 ± 0.5**	**5 ± 1.6**	**4.1 ± 0.3**
9	n-docosane	alkane	25.324	2200	**0.5 ± 0.1**	**0.5 ± 0.1**	**0.6 ± 0.1**	**0.4 ± 0.1**	**0.4 ± 0.1**
10	tricosadiene	alkadiene	26.542	2272	**0.1 ± 0**	**0.1 ± 0.1**	**0.1 ± 0**	**0 ± 0**	**0 ± 0**
11	9-tricosene	alkene	26.589	2274	**1.2 ± 0.1**	**1.4 ± 0.3**	**1.1 ± 0.1**	**1.1 ± 0.2**	**0.9 ± 0.1**
12	7-tricosene	alkene	26.704	2281	**0.2 ± 0**	**0.3 ± 0.1**	**0.2 ± 0**	**0.2 ± 0.1**	**0.2 ± 0**
13	n-tricosane	alkane	27.023	2300	**12.9 ± 1.7**	**11.8 ± 1.2**	**12.9 ± 1.5**	**11.5 ± 1.8**	**9.6 ± 0.8**
14	n-tetracosane	alkane	28.643	2400	**1.6 ± 0.3**	**1.5 ± 0.2**	**1.9 ± 0.4**	**1.4 ± 0.3**	**1.1 ± 0.1**
15	pentacosadiene	alkadiene	29.775	2472	**0.2 ± 0**	**0.3 ± 0**	**0.2 ± 0.1**	**0.2 ± 0.1**	**0.2 ± 0**
16	9-pentacosene	alkene	29.831	2475	**2.1 ± 0.2**	**2.2 ± 0.3**	**1.7 ± 0.2**	**2.1 ± 0.4**	**1.7 ± 0.2**
17	7-pentacosene	alkene	29.945	2482	**0.8 ± 0.1**	**1.1 ± 0.4**	**0.8 ± 0.1**	**1 ± 0.2**	**0.8 ± 0.1**
18	n-pentacosane	alkane	30.223	2500	**20 ± 3.2**	**17.1 ± 1.3**	**21.2 ± 4.9**	**15.8 ± 2.7**	**13.2 ± 0.7**
19	methylpentacosanes	branched alkane	30.736	2534	**0.6 ± 0.1**	**0.6 ± 0.1**	**0.7 ± 0.2**	**0.5 ± 0.1**	**0.4 ± 0**
20	n-hexacosane	alkane	31.724	2600	**2.8 ± 0.7**	**2.1 ± 0.3**	**3.5 ± 1.1**	**2 ± 0.5**	**1.4 ± 0.1**
21	heptacosadiene	alkadiene	32.643	2662	**0.1 ± 0.1**	**0.1 ± 0**	**0.2 ± 0**	**0.1 ± 0**	**0.1 ± 0**
22	9-heptacosene	alkene	32.844	2676	**0.9 ± 0.2**	**0.9 ± 0.2**	**0.6 ± 0.1**	**0.6 ± 0.1**	**0.6 ± 0.1**
23	7-heptacosene	alkene	32.955	2684	**0.3 ± 0.1**	**0.6 ± 0.4**	**0.4 ± 0.1**	**0.3 ± 0.1**	**0.3 ± 0.1**

24	n-heptacosane	alkane	33.195	2700	**53.4 ± 12.4**	**38.2 ± 5.7**	**53.6 ± 13.7**	**34.9 ± 6.4**	**30.4 ± 1.6**
25	methylhetptacosanes	branched alkane	33.641	2732	**2.3 ± 0.4**	**2 ± 0.4**	**1.9 ± 0.2**	**2.3 ± 0.6**	**1.7 ± 0.1**
26	n-octacosane	alkane	34.586	2800	**3.5 ± 1**	**2.2 ± 0.4**	**3.6 ± 1.3**	**1.9 ± 0.5**	**1.6 ± 0.1**
27	nonacosadiene	alkadiene	35.444	2863	**0.2 ± 0.1**	**0.1 ± 0**	**0.2 ± 0.1**	**0.1 ± 0**	**0 ± 0**
28	9-nonacosene	alkene	35.652	2878	**0.5 ± 0.1**	**0.6 ± 0.1**	**0.4 ± 0.1**	**0.3 ± 0.1**	**0.4 ± 0.2**
29	7-nonacosene	alkene	35.708	2882	**0.5 ± 0.1**	**0.5 ± 0.2**	**0.4 ± 0.1**	**0.4 ± 0.1**	**0.4 ± 0.1**
30	n-nonacosane	alkane	35.952	2900	**48.8 ± 10.7**	**35.2 ± 5.8**	**48.4 ± 10.4**	**32.5 ± 5.2**	**30.4 ± 0.9**
31	methylnonacosanes	branched alkane	36.359	2931	**1.5 ± 0.5**	**1.1 ± 0.3**	**1.3 ± 0.1**	**1.1 ± 0.3**	**0.9 ± 0.1**
32	n-triacontane	alkane	37.259	3000	**2.5 ± 0.6**	**1.6 ± 0.3**	**2.5 ± 0.8**	**1.5 ± 0.4**	**4.8 ± 3.7**
33	hentriacontadiene	alkadiene	38.069	3064	**0.3 ± 0.1**	**0.2 ± 0.1**	**0.1 ± 0**	**0.2 ± 0.1**	**0.1 ± 0**
34	9-hentriacontene	alkene	38.241	3077	**3.3 ± 0.7**	**2.7 ± 0.9**	**2.6 ± 0.3**	**2.8 ± 0.8**	**2.8 ± 0.7**
35	7-hentriacontene	alkene	38.328	3084	**4 ± 0.9**	**3.2 ± 0.7**	**3.6 ± 0.4**	**3.4 ± 0.8**	**3.8 ± 1**
36	n-hentriacontane	alkane	38.533	3100	**40.2 ± 7.3**	**28.3 ± 4**	**41.8 ± 7**	**29.5 ± 4.6**	**28.1 ± 2.6**
37	methylhentriacontane	branched alkane	38.901	3115	**0.4 ± 0.1**	**0.3 ± 0.1**	**0.4 ± 0.2**	**0.4 ± 0.1**	**0.5 ± 0.2**
38	tritriacontadiene	alkadiene	40.496	3179	**1.7 ± 0.8**	**1.1 ± 0.4**	**0.9 ± 0.2**	**1.2 ± 0.3**	**1 ± 0.3**
39	X-triacontene	alkene	40.738	3189	**13.5 ± 3.5**	**9.5 ± 2**	**11.3 ± 1.7**	**12.5 ± 3.2**	**10 ± 2**
40	n-tritriacontane	alkane	41.015	3300	**5.6 ± 1.2**	**4.7 ± 1.6**	**5.6 ± 1.3**	**3.5 ± 0.7**	**3.9 ± 0.8**
	Alkanes				**200.7 ± 39.4**	**151 ± 20.8**	**202.7 ± 42.2**	**143.8 ± 25.4**	**132.5 ± 7.6**
	Alkadienes				**2.5 ± 1**	**1.8 ± 0.6**	**1.7 ± 0.4**	**1.8 ± 0.4**	**1.4 ± 0.4**
	Alkene				**27.4 ± 5.5**	**23.1 ± 5.2**	**23 ± 3.1**	**24.8 ± 6**	**21.9 ± 4.3**
	Branched alkanes				**4.8 ± 1**	**4.1 ± 0.9**	**4.1 ± 0.7**	**4.3 ± 1.1**	**3.5 ± 0.4**
	NI				**6.5 ± 0.8**	**7.4 ± 1**	**7.6 ± 1.4**	**7.5 ± 1.6**	**6.3 ± 1.2**

Table S3. Suppl. Material: CHC from honeybees in Experiment III.

Compound No	Compund ID	Compound Class	Retention Time	Retention index	CTRL (ug/bee, D)	INF (ug/bee, D+N)	CTRL+COUM (ug/bee, C)	INF+COUM (ug/bee, C+N2)
1	IS (tridecane)	IS	8.934	1300				
2	NI	NI	12.138	1476	0.2 ± 0.1	0.3 ± 0.1	0.3 ± 0.1	0.3 ± 0.1
3	NI	NI	12.96	1521	0.3 ± 0	0.2 ± 0.1	0.3 ± 0.1	0.3 ± 0.1

4	NI	NI	15.994	1687	0.3 ± 0	0.4 ± 0.1	0.4 ± 0.1	0.3 ± 0.1
5	n-nonadecane	alkane	19.874	1900	1.9 ± 0.3	1.8 ± 0.5	2.3 ± 0.5	1.5 ± 0.4
6	NI	NI	20.351	1937	0.1 ± 0	0.1 ± 0	0.1 ± 0	0.1 ± 0
7	n-eicosane	alkane	21.147	2000	0.1 ± 0	0.1 ± 0	0.1 ± 0	0.1 ± 0
8	n-heneicosane	alkane	23.572	2100	4.1 ± 0.4	3 ± 0.7	4.7 ± 1	2.9 ± 0.8
9	n-docosane	alkane	25.324	2200	0.7 ± 0.2	0.4 ± 0.1	0.5 ± 0.1	0.5 ± 0.1
10	tricosadiene	alkadiene	26.542	2272	0.1 ± 0	0.2 ± 0.1	0.1 ± 0	0.3 ± 0.1
11	9-tricosene	alkene	26.589	2274	5.1 ± 2.7	2.4 ± 0.6	2.4 ± 0.4	2.6 ± 0.9
12	7-tricosene	alkene	26.704	2281	0.4 ± 0.1	0.3 ± 0.1	0.4 ± 0.1	0.3 ± 0.1
13	n-tricosane	alkane	27.023	2300	17.4 ± 2.2	17 ± 2.8	17.5 ± 2	16.1 ± 4
14	n-tetracosane	alkane	28.643	2400	1.1 ± 0.1	1.3 ± 0.3	1.3 ± 0.2	1.3 ± 0.3
15	pentacosadiene	alkadialkene	29.775	2472	0.6 ± 0.2	0.8 ± 0.3	0.8 ± 0.2	0.9 ± 0.4
16	9-pentacosene	alkene	29.831	2475	4.6 ± 0.7	4.6 ± 1	4.4 ± 0.5	4.4 ± 1.2
17	7-pentacosene	alkene	29.945	2482	1.4 ± 0.2	2 ± 0.5	1.5 ± 0.2	2.1 ± 0.6
18	n-pentacosane	alkane	30.223	2500	21.3 ± 1.9	23.8 ± 2.9	21.7 ± 2.5	21.1 ± 4.4
19	methylpentacosanes	branched alkane	30.736	2534	1 ± 0.2	1.1 ± 0.3	1.2 ± 0.3	1.1 ± 0.4
20	n-hexacosane	alkane	31.724	2600	1.3 ± 0.2	1.7 ± 0.5	1.5 ± 0.3	1.5 ± 0.4
21	heptacosadiene	alkadiene	32.643	2662	0.2 ± 0	0.1 ± 0	0.2 ± 0	0.2 ± 0
22	9-heptacosene	alkene	32.844	2676	2.9 ± 0.5	3.2 ± 0.8	3.1 ± 0.6	2.8 ± 0.8
23	7-heptacosene	alkene	32.955	2684	0.8 ± 0.1	1.1 ± 0.3	1 ± 0.2	0.9 ± 0.3
24	n-heptacosane	alkane	33.195	2700	22.9 ± 1.9	24.8 ± 3.6	22.4 ± 2.4	21.8 ± 4.6
25	methylhetptacosanes	branched alkane	33.641	2732	4.3 ± 0.6	5 ± 1.2	5.2 ± 0.9	4.6 ± 1.4
26	n-octacosane	alkane	34.586	2800	0.6 ± 0.1	0.7 ± 0.2	0.8 ± 0.1	0.7 ± 0.2
27	nonacosadiene	alkadiene	35.444	2863	2.2 ± 0.4	1.5 ± 0.4	1.4 ± 0.3	1.6 ± 0.7
28	9-nonacosene	alkene	35.652	2878	2.1 ± 0.3	2.2 ± 0.6	1.8 ± 0.3	2.1 ± 0.7
29	7-nonacosene	alkene	35.708	2882	0.8 ± 0.1	0.9 ± 0.2	0.9 ± 0.1	0.7 ± 0.2
30	n-nonacosane	alkane	35.952	2900	9 ± 1.1	10.4 ± 2.1	9.9 ± 1	8.9 ± 2.4
31	methylnonacosanes	branched alkane	36.359	2931	2.4 ± 0.3	2.8 ± 0.7	2.8 ± 0.5	2.6 ± 0.8

32	n-triacontane	alkane	37.259	3000	0.2 ± 0	0.2 ± 0.1	0.2 ± 0	0.2 ± 0.1
33	hentriacontadiene	alkadiene	38.069	3064	0.2 ± 0	0.2 ± 0.1	0.2 ± 0	0.5 ± 0.3
34	9-hentriacontene	alkene	38.241	3077	2.9 ± 0.5	3.2 ± 0.9	3.1 ± 0.5	2.7 ± 0.8
35	7-hentriacontene	alkene	38.328	3084	2.6 ± 0.4	3.2 ± 0.8	2.8 ± 0.3	2.7 ± 0.7
36	n-hentriacontane	alkane	38.533	3100	3.9 ± 0.7	5.5 ± 1.4	4 ± 0.6	3.6 ± 1.4
37	methylhentriacontane	branched alkane	38.901	3115	0.6 ± 0.1	0.8 ± 0.2	0.7 ± 0.1	0.7 ± 0.2
38	tritriacontadiene	alkadiene	40.496	3179	0.5 ± 0.1	0.9 ± 0.3	0.5 ± 0.1	1.3 ± 0.6
39	X-triacontene	alkene	40.738	3189	5.5 ± 0.8	7.1 ± 1.7	5.6 ± 0.5	5.2 ± 2.1
40	n-tritriacontane	alkane	41.015	3300	0.3 ± 0.1	0.5 ± 0.1	0.3 ± 0	0.3 ± 0.1
	Alkanes				68 ± 18	91.3 ± 13.9	87.3 ± 9.1	80.5 ± 19
	Alkadienes				3 ± 0.9	3.8 ± 1	3.2 ± 0.5	4.8 ± 1.5
	Alkene				21.9 ± 6.1	30.2 ± 7.1	26.8 ± 2.6	26.5 ± 8.1
	Branched alkanes				6.8 ± 2	9.7 ± 2.4	9.8 ± 1.7	8.9 ± 2.8
	NI				0.7 ± 0.2	0.9 ± 0.1	1 ± 0.2	1 ± 0.1

Figure S1. CHC profiles principal component analyses (PCA) run on scaled and centered data for experiment I (**A**), experiment II (**B**) and experiment III (**C**).

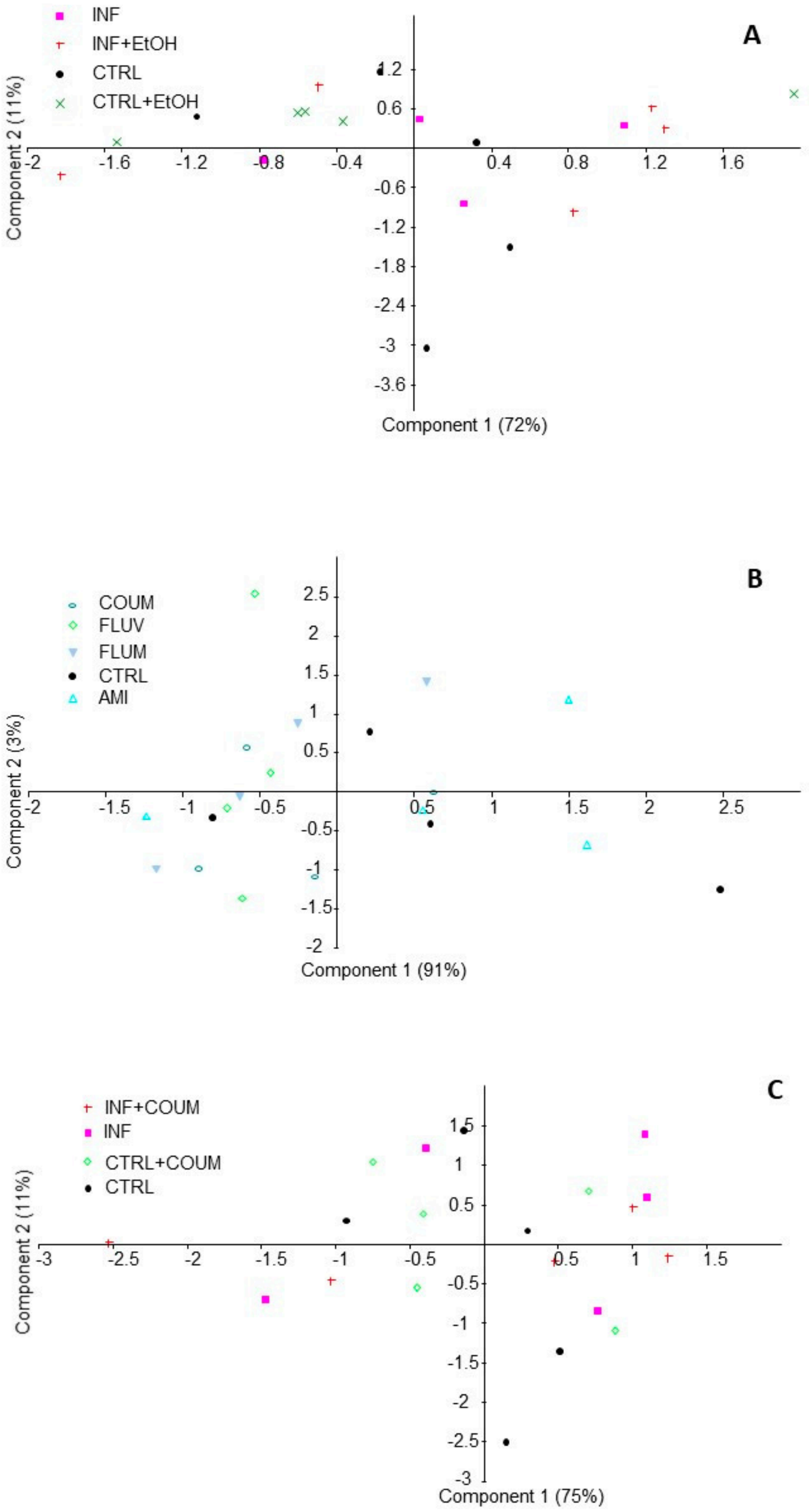

Supplement: Supplementary file 1 [file vetsci-07-00199-s001.pdf]
